# Comparative Effectiveness of New Approaches to Improve Mortality Risk Models From Medicare Claims Data

**DOI:** 10.1001/jamanetworkopen.2019.7314

**Published:** 2019-07-17

**Authors:** Harlan M. Krumholz, Andreas C. Coppi, Frederick Warner, Elizabeth W. Triche, Shu-Xia Li, Shiwani Mahajan, Yixin Li, Susannah M. Bernheim, Jacqueline Grady, Karen Dorsey, Zhenqiu Lin, Sharon-Lise T. Normand

**Affiliations:** 1 Center for Outcomes Research and Evaluation, Yale New Haven Hospital, New Haven, Connecticut; 2 Section of Cardiovascular Medicine, Department of Internal Medicine, Yale School of Medicine, New Haven, Connecticut; 3 Department of Health Policy and Management, Yale School of Public Health, New Haven, Connecticut; 4 Section of General Internal Medicine, Department of Internal Medicine, Yale School of Medicine, New Haven, Connecticut; 5 Section of General Pediatrics, Department of Pediatrics, Yale School of Medicine, New Haven, Connecticut; 6 Department of Biostatistics, Harvard T.H. Chan School of Public Health, Harvard University, Boston, Massachusetts; 7 Department of Health Care Policy, Harvard Medical School, Boston, Massachusetts

## Abstract

**Importance:**

Risk adjustment models using claims-based data are central in evaluating health care performance. Although US Centers for Medicare & Medicaid Services (CMS) models apply well-vetted statistical approaches, recent changes in the *International Classification of Diseases, Ninth Revision, Clinical Modification* (*ICD-9-CM*) coding system and advances in computational capabilities may provide an opportunity for enhancement.

**Objective:**

To examine whether changes using already available data would enhance risk models and yield greater discrimination in hospital-level performance measures.

**Design, Setting, and Participants:**

This comparative effectiveness study used *ICD-9-CM* codes from all Medicare fee-for-service beneficiary claims for hospitalizations for acute myocardial infarction (AMI), heart failure (HF), or pneumonia among patients 65 years and older from July 1, 2013, through September 30, 2015. Changes to current CMS mortality risk models were applied incrementally to patient-level models, and the best model was tested on hospital performance measures to model 30-day mortality. Analyses were conducted from April 19, 2018, to September 19, 2018.

**Main Outcomes and Measures:**

The main outcome was all-cause death within 30 days of hospitalization for AMI, HF, or pneumonia, examined using 3 changes to current CMS mortality risk models: (1) incorporating present on admission coding to better exclude potential complications of care, (2) separating index admission diagnoses from those of the 12-month history, and (3) using ungrouped *ICD-9-CM* codes.

**Results:**

There were 361 175 hospital admissions (mean [SD] age, 78.6 [8.4] years; 189 225 [52.4%] men) for AMI, 716 790 hospital admissions (mean [SD] age, 81.1 [8.4] years; 326 825 [45.6%] men) for HF, and 988 225 hospital admissions (mean [SD] age, 80.7 [8.6] years; 460 761 [46.6%] men) for pneumonia during the study; mean 30-day mortality rates were 13.8% for AMI, 12.1% for HF, and 16.1% for pneumonia. Each change to the models was associated with incremental gains in C statistics. The best model, incorporating all changes, was associated with significantly improved patient-level C statistics, from 0.720 to 0.826 for AMI, 0.685 to 0.776 for HF, and 0.715 to 0.804 for pneumonia. Compared with current CMS models, the best model produced wider predicted probabilities with better calibration and Brier scores. Hospital risk-standardized mortality rates had wider distributions, with more hospitals identified as good or bad performance outliers.

**Conclusions and Relevance:**

Incorporating present on admission coding and using ungrouped index and historical *ICD-9-CM* codes were associated with improved patient-level and hospital-level risk models for mortality compared with the current CMS models for all 3 conditions.

## Key Points

**Question:**

Could present on admission indicators and ungrouped diagnostic codes enhance risk models for acute myocardial infarction, heart failure, and pneumonia mortality measures and improve discrimination of hospital-level performance?

**Findings:**

In this comparative effectiveness study including all Medicare fee-for-service beneficiaries hospitalized for acute myocardial infarction, heart failure, or pneumonia at acute care hospitals, incorporating present on admission coding and ungrouped historical and index admission *International Classification of Diseases, Ninth Revision, Clinical Modification* codes was associated with greater discrimination in patient-level and hospital-level 30-day mortality risk models.

**Meaning:**

Changes incurring no additional cost could enhance the risk adjustment for mortality and increase discrimination of hospital-level performance.

## Teaser

This comparative effectiveness study examines current US Centers for Medicare & Medicaid Services 30-day mortality risk models vs novel risk models for acute myocardial infarction, heart failure, and pneumonia hospitalizations.

## Introduction

Risk models using administrative claims–based data play a central role in evaluating health care performance, setting payments, and conducting research.^1,2,3,4,5,6,7^ We hypothesized that 2 approaches could improve the performance of these models. First, the models could potentially improve by using present on admission (POA) codes. In 2014, the US Centers for Medicare & Medicaid Services (CMS) began mandating hospitals to add POA designations, which denoted conditions that predated the hospitalization.^8^ Many diagnosis codes used exclusively on the index hospitalization were excluded from models predicting outcomes. For example, the CMS performance models excluded diagnoses because they might have represented complications associated with clinical quality. The use of these codes with the knowledge that they were present on admission would increase the number of codes available for risk adjustment. Second, claims-based models often combine diagnoses into clinically coherent groups to reduce the number of variables. For example, CMS bases many of its models on modifications of the hierarchical condition categories (HCCs) to group codes.^9,10^ For example, in the CMS mortality model, the codes for historical diagnoses and procedures from the previous 12 months are combined with codes from the index admission into 1 set of risk variables. A potential limitation is that codes that comprise a group may have different associations with the outcome but the group’s overall effect is a weighted mean. Also, the effect of lower-frequency codes could be overwhelmed by that of higher-frequency codes. Advancements in computational capabilities and analytical methods enable us to handle much larger amounts of information efficiently and provide an opportunity to consider risk variables using ungrouped codes.^11^

Accordingly, we tested whether leveraging POA coding and using individual codes rather than grouped codes could be associated with improved model performance. These changes use data already available in claims and incur no additional marginal cost. Specifically, we explored 3 changes to current patient-level risk models: (1) incorporating POA coding to distinguish conditions present at the time of admission from those emerging during hospitalization, (2) separating POA diagnosis codes from those present in encounters during the prior 12 months, and (3) disaggregating codes currently used in the risk variable groupers. The best-performing models were compared with publicly reported CMS hospital performance measures. This assessment focused on the publicly reported 30-day mortality measures for acute myocardial infarction (AMI), heart failure (HF), and pneumonia.

## Methods

### Data Source and Study Sample

We applied the cohort definitions for the CMS 30-day all-cause mortality measures after hospitalization for AMI,^5^ HF,^6^ or pneumonia.^12^ Medicare Standard Analytic and denominator files identified all hospitalizations at acute care hospitals with a principal discharge diagnosis of AMI, HF, or pneumonia from July 1, 2013, through September 30, 2015. We defined cohorts with the same *International Classification of Diseases, Ninth Revision, Clinical Modification* (*ICD-9-CM*)^13^ codes used in the CMS publicly reported mortality measures. We chose to focus on a period exclusively using *ICD-9-CM* codes because it was not practical to combine both *ICD-9-CM* and *International Statistical Classification of Diseases, Tenth Revision, Clinical Modification (ICD-10-CM)* codes in the same models. We did not have enough data coded in *ICD-10-CM* to conduct these analyses exclusively in the *ICD-10-CM* data. We included hospitalizations for patients 65 years or older. We excluded hospitalizations from which patients were discharged against medical advice and for patients with less than 1 year of prior enrollment in Medicare fee-for-service. We further excluded records for which POA coding was missing for the principal diagnosis of the index admission. We linked transfers into a single episode of care and assigned patients to the index admitting hospital. We used Medicare claims for 12 months before the index admission. The Human Investigation Committee at Yale University approved an exemption for this study to use CMS claims and enrollment data and waived the requirement for informed consent because the research involved no more than minimal risk and could not be practicably carried out without the waiver. Data analyses were conducted from April 19, 2018, to September 19, 2018.

### Outcome

The outcome was death from any cause within 30 days of the hospital admission date for AMI, HF, and pneumonia. Death date was identified by data in CMS enrollment files or from inpatient claim discharge status for death during the index hospitalization.

### Model Modifications

We tested 3 changes to current risk models: (1) incorporating POA coding to better distinguish conditions that were POA from those that were complications of care, (2) separating diagnosis codes in the index admission from those coded within the prior 12 months, and (3) disaggregating codes within risk variable groupers and using individual *ICD-9-CM* codes instead. The changes were applied incrementally to patient-level models, with the best model then tested at the hospital level. To develop and test the potential changes, we used 5-fold cross-validation to control for model overfitting, with each iteration training generalized linear models (GLMs) on 80% of the cohort and then testing on the remaining 20%.

#### Incorporating POA Coding

Present on admission coding identifies conditions present at admission. The current CMS mortality measures’ risk models, developed before the use of POA indicators, exclude specific diagnoses coded in the index admission that could represent complications of care.^14^ We tested whether incorporating POA coding into the CMS model could be associated with improved patient-level mortality model discrimination. The official CMS POA exempt list was reviewed by a panel of clinical experts, and conditions that were judged to have any significant potential to be a complication of care were removed (eMethods in the Supplement). For example, code V66.7 (encounter for palliative care) was in the 2016 CMS POA exempt list but we removed it from the clinically vetted modified list.

#### Separating Historical Diagnosis Codes From Index Admission Codes

In the CMS model, each grouper-based variable indicates whether at least 1 diagnosis code of a category was found in claims during 12 months before admission or within the index admission. We tested whether discrimination improved when separating the historical conditions from the POA conditions of the index admission, treating them as distinct variables.

#### Disaggregating Risk Variable Groupers

We incrementally compared patient-level mortality discrimination among risk variables of different types. First, we used the CMS risk variables. The risk variables of the publicly reported measure models use modified condition categories (MCCs) to capture patient-level severity and to reduce dimensionality and 4 non–diagnosis-based variables (age, sex, history of percutaneous coronary intervention, and history of coronary artery bypass). The MCCs were constructed from the 201 condition categories (CCs) that are the bottom level of the version 22 HCCs using previously established methods.^10^ Modified condition categories may include individual CCs, groups of CCs, or subsets of codes from within CCs. For example, in the AMI model, the diabetes variable consists of any *ICD-9-CM* code within CC 17, CC 18, CC 19, or CC 123.

Next, we considered using the full set of CCs. To incrementally explore using more disaggregated groupers, we replaced the current MCCs with all 201 individual CCs. Finally, we investigated using individual *ICD-9-CM* codes, eliminating the use of a grouper. We replaced the MCCs with a subset of individual *ICD-9-CM* codes as risk variables. We restricted index admission *ICD-9-CM* codes to those that were identified as POA or those that were not explicitly identified as not POA while also on the modification of CMS’s POA-exempt list. Also, we considered all individual *ICD-9-CM* codes in claims of the 12-month history, pooling together inpatient, outpatient, and Part B settings. For CMS risk models, any groupers or risk factors with less than 1% frequency among all admissions are considered low frequency. For our study, we restricted to codes with a frequency of more than 0.5%, keeping index and history *ICD-9-CM* codes separate.

### Statistical Analysis

We incrementally incorporated the 3 proposed model changes in risk-adjusted GLMs at the patient level with the goal of choosing the best one to explore hospital-level performance within the practical constraints of fitting hospital-level hierarchical GLMs (HGLMs), given the large number of hospitals and admission records. All the GLM and HGLM models use a binary distribution and a logit link function. In our experience, these hospital-level HGLMs need to be limited to a maximum of approximately 200 variables with low associated *P* values to reliably converge and be practical for the time-consuming bootstrapping required in CMS methodology. Hence, with the end goal of demonstrating a better separation of performance of the hospitals, we limited the modified patient-level models tested at the hospital level to 200 or fewer variables. The likelihood-ratio test was used to find P values. To further improve HGLM convergence issues, we required variables to have a 2-tailed *P* value less than .005 in the patient-level GLM.

To meet these requirements with a model based on individual *ICD-9-CM* codes, we used a basic variable-selection strategy. After excluding codes with less than 0.5% frequency, we used a least absolute shrinkage and selection operator parameter grid search to select the best set of variables, with total count not exceeding 200, from the combined set of index admission *ICD-9-CM* codes, history *ICD-9-CM* codes, and non–diagnosis-based variables in the CMS models. Next, a GLM was fit using these selected codes, and only those with a *P* value less than .005 were retained for the final model, hereafter the *individual-codes model* (eFigure 1 in the Supplement). Whenever 5-fold cross-validation was used, we only used the training set for code selection. The discrimination performance of each of the previously described model modifications was compared using the C statistic, testing the statistical significance of the differences using the method of DeLong et al.^15^

We compared patient-level performance of the individual-codes model to CMS risk adjustment using the C statistic and the Brier score. Following CMS methodology, models were trained and tested on the full cohorts. The Brier score can be considered a measure of the calibration of a prediction model (lower is better) and is a combination of several components, including reliability, a measure of the error in a calibration curve (lower is better), and resolution, reflecting how well a model separates predictions from the mean event rate (higher is better). We also compared the calibration slope (closer to 1 is better) and predictive range (wider is better) across both models. We included additional model comparisons, such as receiver operating characteristic curves, calibration plots (eFigure 2 in the Supplement), log odds plots (eFigure 3 in the Supplement), and shift tables (eTable 1 in the Supplement), and listed the top codes that each individual-codes model selected (eTables 2, 3, and 4 in the Supplement).

To assess the association of the proposed changes to patient-level risk adjustment with hospital performance profiling, we used the CMS HGLM approach to calculate the hospital risk-standardized mortality rates (RSMRs).^16,17^ We compared hospital-level performance using the CMS models with those incorporating the risk factors of the individual-codes models. The individual-codes models included 150, 182, and 186 variables for the AMI, HF, and pneumonia condition cohorts, respectively. We compared the distributions and between-hospital variances of RSMRs. A larger SD, interquartile range (IQR), and range would indicate that the RSMRs of different hospitals are spread out wider and thus more distinguishable. We used the *F* test (number of hospitals as degree of freedom for both numerator and denominator) to examine whether between-hospital variances from HGLM models were equal. We used folded *F* tests to compare variances of the RSMR distributions. We also calculated the weighted Pearson correlation coefficient between the RSMRs using the CMS model and the individual-codes model, using the inverse of the variance of *ICD-9-CM* RSMRs as weights.^18,19^ Hospital RSMR variances were calculated using bootstrap methods defined in CMS public reporting.^14^ We used 5000 bootstraps for each condition for the CMS and individual-codes models.

On its public Hospital Compare website,^20^ CMS reports annually on hospitals with at least 25 cases of AMI, HF, or pneumonia, using the 3 performance categories of no different than the national rate, better than the national rate, or worse than the national rate. In addition to point estimate and CI, we classified hospitals into these categories by whether RSMR CIs contained the national rate.^14^ We compared the frequency of hospitals in each category and studied the hospital category shifts between the CMS and individual-codes model.

All GLM-related analyses were conducted in R statistical software version 3.3 (R Project for Statistical Computing) using the stats package or in Python version 2.7 (Python Software Foundation) using the scikit-learn or StatsModels packages. Our least absolute shrinkage and selection operator analysis was implemented using the GLMNET library in both R and Python. All hospital-level analyses were performed using SAS statistical software version 9.4 (SAS Institute).

## Results

### Cohort Description

The AMI, HF, and pneumonia cohorts are described in Table 1. There were 361 175 hospital admissions (mean [SD] age, 78.6 [8.4] years; 189 225 [52.4%] men) for AMI, 716 790 hospital admissions (mean [SD] age, 81.1 [8.4] years; 326 825 [45.6%] men) for HF, and 988 225 hospital admissions (mean [SD] age, 80.7 [8.6] years; 460 761 [46.6%] men) for pneumonia. The mean 30-day mortality rates were 13.8% for AMI, 12.1% for HF, and 16.1% for pneumonia.

**Table 1.** Cohort Description for Acute Myocardial Infarction, Heart Failure, and Pneumonia

Variable	Acute Myocardial Infarction	Heart Failure	Pneumonia
Total index admissions, No.	361 175	716 790	988 225
Unique patients, No. (%)	352 457 (97.6)	644 883 (90.0)	926 511 (93.8)
Unique hospitals, No.	4036	4380	4462
Observed 30-d mortality rate, %	13.8	12.1	16.1
Men, No. (%)	189 225 (52.4)	326 825 (45.6)	460 761 (46.6)
Age, mean (SD), y^a^	78.6 (8.4)	81.1 (8.4)	80.7 (8.6)
Race/ethnicity, No. (%)			
White, non-Hispanic	313 942 (86.9)	602 993 (84.1)	862 175 (87.2)
African American	28 716 (8.0)	80 665 (11.3)	74 085 (7.5)
Hispanic	5548 (1.5)	11 913 (1.7)	17 373 (1.8)
Asian	5104 (1.4)	9024 (1.3)	15 495 (1.6)
North American Native	2080 (0.6)	3690 (0.5)	6640 (0.7)
Other	4286 (1.2)	6830 (1.0)	9959 (1.0)
Unknown	1499 (0.4)	1675 (0.2)	2497 (0.3)

^a^ Only patients 65 years and older were included.

### Performance of Patient-Level 30-Day Mortality Models

Each additional change to the models resulted in incremental gains in C statistics (Table 2). The highest overall gains were achieved by the combination of changes incorporated in the individual-codes models, improving the CMS C statistics from 0.720 to 0.826 for AMI (*P* < .001), 0.685 to 0.776 for HF (*P* < .001), and 0.715 to 0.804 for pneumonia (*P* < .001).

**Table 2.** C Statistics of Patient-Level CMS Models Without POA Codes and Proposed Models^a^^,^^b^

Model	Acute Myocardial Infarction Mortality		Heart Failure Mortality		Pneumonia Mortality	
	Mean Variables, No.	C Statistic (SD)	Mean Variables, No.	C Statistic (SD)	Mean Variables, No.	C Statistic (SD)
Current CMS MCC model without incorporating POA codes and with pooled index and history CCs^c^	27	0.720 (0.004)	24	0.685 (0.005)	36	0.715 (0.004)
CMS MCC model incorporating POA codes with pooled index and history CCs	27	0.768 (0.004)	24	0.694 (0.004)	36	0.735 (0.005)
CMS MCC model incorporating POA codes and separating index CCs from history CCs	48	0.783 (0.004)	44	0.710 (0.005)	68	0.755 (0.006)
Full HCC model incorporating POA codes with index HCCs only	207	0.807 (0.004)	205	0.744 (0.006)	205	0.773 (0.006)
Full HCC model incorporating POA codes and separating index HCCs from history HCCs	408	0.810 (0.004)	406	0.756 (0.005)	406	0.783 (0.006)
Individual-codes model incorporating POA codes with codes selected from a pool of separated index and history *ICD* codes, mean (SD)	145.8 (3.3)	0.826 (0.004)	172.0 (7.5)	0.776 (0.005)	182.4 (6.5)	0.804 (0.006)

Abbreviations: CC, condition category; CMS, Centers for Medicare & Medicaid Services; HCC, hierarchical condition categories; *ICD*, *International Classification of Diseases*; MCC, modified condition category; POA, present on admission.

^a^ Patient-level models were calculated using logistic regression.

^b^ Incorporates POA codes, separates history from index risk variables, and assesses different types of diagnosis-based risk variables for acute myocardial infarction, heart failure, and pneumonia 30-day mortality measures. (Results presented are the mean through 5-fold cross-validation.) All models also adjust for age, sex, and history of coronary artery bypass grafting or percutaneous coronary intervention.

^c^ Used current CMS algorithm to distinguish conditions POA from complications of care.

### Comparison of CMS and Individual-Codes Patient-Level Models for 30-Day Mortality 

Table 3 compares risk predictions and metrics of the patient-level CMS and individual-codes models. For these results, we did not use the 5-fold cross-validation used in Table 2 because we were comparing with the CMS model as implemented, which, following CMS methodology,^14^ is trained and tested on the full cohort. Using the individual index POA and history codes selected by our method (150 for AMI, 182 for HF, and 186 for pneumonia) was associated with substantially improved model performance compared with the CMS risk model for all 3 mortality measures. We found significantly higher C statistics for the individual-codes models for all 3 conditions, increasing from 0.720 to 0.828 for AMI, 0.685 to 0.778 for HF, and 0.715 to 0.805 for pneumonia. Brier scores for the individual-codes models were 0.092 for AMI, 0.102 for HF, and 0.111 for pneumonia; for the CMS models, the Brier scores were 0.110 for AMI, 0.102 for HF, and 0.126 for pneumonia. Resolution scores were 0.027 for AMI, 0.013 for HF, and 0.025 for pneumonia for individual-codes models and 0.0086 for AMI, 0.0044 for HF, and 0.001 for pneumonia for the CMS models. The 2 models had similar reliability for all 3 conditions.

**Table 3.** Comparison of Performance Metrics of Patient-Level CMS and Individual-Codes Risk Models for Acute Myocardial Infarction, Heart Failure, and Pneumonia 30-Day Mortality Measures^a^

Measure	Acute Myocardial Infarction		Heart Failure		Pneumonia	
	CMS Model	Individual-Codes Model^b^	CMS Model	Individual-Codes Model^b^	CMS Model	Individual-Codes Model^b^
C statistic	0.720	0.828	0.685	0.778	0.715	0.805
Slope of calibration	0.990	0.995	0.994	0.998	0.976	0.989
Brier score (SD)^c^	0.110 (0.00044)	0.092 (0.00049)	0.102 (0.00030)	0.093 (0.00032)	0.126 (0.00026)	0.111 (0.00029)
Reliability (SD)^c^	0.00021 (0.000022)	0.00017 (0.000017)	0.00016 (0.000013)	0.00025 (0.000017)	0.00050 (0.000020)	0.00050 (0.000020)
Resolution (SD)^d^	0.0086 (0.00013)	0.027 (0.00025)	0.0044 (0.000059)	0.013 (0.00012)	0.0099 (0.000080)	0.025 (0.00014)
Predicted probability, mean (SD) [range]	0.138 (0.099) [0.019-0.850]	0.138 (0.169) [0.003-0.999]	0.121 (0.073) [0.015-0.724]	0.121 (0.122) [0.001-0.985]	0.161 (0.106) [0.016-0.852]	0.161 (0.163) [0.002-0.996]

Abbreviation: CMS, Centers for Medicare & Medicaid Services.

^a^ Models were trained and tested on the full cohorts.

^b^ The individual-codes model used 150 variables for acute myocardial infarction, 182 variables for heart failure, and 186 variables for pneumonia.

^c^ For Brier score and reliability, lower is better.

^d^ For resolution, higher is better.

The range of predicted probabilities of 30-day mortality was wider for the individual-codes models than for the CMS models (AMI, 0.003-0.999 vs 0.019-0.850; HF, 0.001-0.985 vs 0.015-0.724; pneumonia, 0.002-0.996 vs 0.016-0.852). Standard deviations of the predicted probabilities were also higher for the individual-codes models than for CMS models (AMI, 0.169 vs 0.099; HF, 0.122 vs 0.073; pneumonia, 0.163 vs 0.106).

### Performance of Hospital-Level HGLM Models

A total of 4036 hospitals contributed claims for the AMI cohort, 4380 hospitals contributed claims for HF, and 4462 hospitals contributed claims for pneumonia. Of these, there were at least 25 cases at 2218 hospitals for AMI, 3323 hospitals for HF, and 3877 hospitals for pneumonia.

The RSMRs calculated using individual-codes models had a wider distribution compared with those using CMS models for HF and pneumonia measures (*t* test comparing the RSMRs from the 2 models had *P* < .001), while the AMI RSMR distribution appeared to be wider but not statistically significant (Table 4; eFigure 4 in the Supplement). The between-hospital variances were higher in HGLMs using individual-codes models compared with CMS models, increasing from 0.035 to 0.047 for AMI (*P* < .001), 0.051 to 0.071 for HF (*P* < .001), and 0.048 to 0.180 for pneumonia (*P* < .001). The SDs, IQRs, and ranges for RSMRs using individual-codes models were also higher for all 3 conditions (Table 4). The model for AMI had the smallest change, while the model for pneumonia had the largest change, and its IQR almost doubled from 2.4 percentage points (IQR, 15.0%-17.4%) to 4.0 percentage points (IQR, 14.2%-18.3%). The weighted Pearson correlations between RSMRs for CMS models and individual-codes models were 0.83 for AMI, 0.83 for HF, and 0.73 for pneumonia.

**Table 4.** Distribution of Hospital-Level Risk-Standardized Mortality Rates Comparing Publicly Reported CMS Models With Individual-Codes Models Incorporating Proposed Changes for Acute Myocardial Infarction, Heart Failure, and Pneumonia 30-Day All-Cause Mortality Measures Among Hospitals With at Least 25 Cases

Measure	Risk-Standardized Mortality Rate					
	Acute Myocardial Infarction (n = 2218)		Heart Failure (n = 3323)		Pneumonia (n = 3877)	
	CMS Model	Individual-Codes Model	CMS Model	Individual-Codes Model	CMS Model	Individual-Codes Model
Mean (SD)	13.7 (1.1)	13.8 (1.1)	12.1 (1.5)	12.1 (1.7)	16.2 (1.9)	16.5 (3.3)
Median (IQR) [range]	13.6 (13.0-14.4) [9.6-18.1]	13.7 (13.0-14.4) [9.5-19.2]	12.1 (11.1-13.1) [6.5-17.9]	12.0 (11.0-13.2) [6.6-19.7]	16.1 (15.0-17.4) [9.4-26.4]	16.1 (14.2-18.3) [8.1-34.2]
IQR difference	1.4	1.4	2.0	2.2	2.4	4.0
Range difference	8.5	9.7	11.4	13.1	17.0	26.1

Abbreviations: CMS, Centers for Medicare & Medicaid Services; IQR, interquartile range.

When comparing performance categories assigned by the 2 models (Table 5), the individual-codes model identified more hospitals as better than or worse than the national mean for all 3 conditions. The extent of variation between models was different for all 3 conditions, with pneumonia having the largest increase. For example, using the individual-codes model compared with the CMS model, we identified 34 (1.5%) vs 30 hospitals (1.4%) as better than the national rate for AMI mortality, 185 (5.6%) vs 124 hospitals (3.7%) as better than the national rate for HF mortality, and 492 (12.7%) vs 192 hospitals (5.0%) as better than the national rate for pneumonia mortality. Similar differences were noted for hospitals identified as worse than the national rate. The shift in the performance categories using each model is shown in eTable 5 in the Supplement. Most of the better and worse outliers reported by CMS remained in the same categories when assigned by individual-codes models.

**Table 5.** Distribution of Hospital-Level Performance Categories Comparing Publicly Reported CMS Models With Individual-Codes Models Incorporating Proposed Patient-Level Model Changes to Acute Myocardial Infarction, Heart Failure, and Pneumonia 30-Day All-Cause Mortality Measures Among Hospitals With at Least 25 Cases

Condition	No. (%)		
	Better Than National Rate	No Different Than National Rate	Worse Than National Rate
Acute myocardial infarction mortality			
CMS model	30 (1.4)	2175 (98.1)	13 (0.6)
Individual-codes model	34 (1.5)	2158 (97.3)	26 (1.2)
Heart failure mortality			
CMS model	124 (3.7)	3139 (94.5)	60 (1.8)
Individual-codes model	185 (5.6)	3037 (92.0)	81 (2.4)
Pneumonia mortality			
CMS model	192 (5.0)	3500 (90.3)	185 (4.8)
Individual-codes model	492 (12.7)	3077 (79.4)	308 (7.9)

Abbreviation: CMS, Centers for Medicare & Medicaid Services.

## Discussion

We introduce a new approach to the risk adjustment used in conducting research and profiling hospital mortality performance. The proposed methods use data already available in claims and therefore incur no additional marginal cost to include in risk models. By leveraging the introduction of POA coding, separating codes first noted on the index admission from those of the prior year, and disaggregating previously grouped codes, we markedly improved the discrimination of the risk-adjusted models for 30-day AMI, HF, and pneumonia mortality. In hospital profiling, this approach allowed us to detect more variation, identifying many more hospitals as better or worse than the national mean, which is important because lack of variation in performance has been identified as a problem with the current measures.^21^

The improvement associated with the use of POA coding would be easy to implement. For example, CMS 30-day mortality outcome measures, developed before POA information was reliably available, exclude a list of diagnoses that have the potential to be complications of care but could also convey valuable information about preadmission risk. Using the POA coding that is now available yields substantially improved patient-level model performance across all 3 conditions. Also, separating historical and index diagnoses was associated with model improvement. Incorporating these changes into the risk models is not difficult. Experiments using custom, data-driven groupers showed some improvement over traditional groupers, with better results as the number of categories increased and the sizes of the individual categories decreased. However, the most substantial improvement was associated with replacing risk factors based on traditional groupers with a larger number of variables consisting of individual diagnosis codes. Groupers may obscure low-frequency codes and the heterogeneity of effect among the constituent codes.

There are several implications of the findings of our approach to use ungrouped diagnostic codes with their associated POA designations and index or prior timing. They provide a rationale for revisiting the prior assumptions about our approach to risk adjustment for outcomes performance models. Groupers have been used to efficiently handle extremely large sets of variables and simplify the computations to model them, but we found that the grouping of variables may not be necessary owing to the availability of computationally advanced algorithms. Many stakeholders potentially benefit from more accurate measures.

Although examination of improvements in measure score validity resulting from these methods was beyond the scope of this study, the gains demonstrated in discrimination of the patient-level models could translate into more precise characterization of hospitals’ performance. More investigation is required to reach such a conclusion. However, because these measures are currently used to profile hospital quality and to assess payments for better or worse performance, there would be a clear benefit to patients and hospitals if gains in the performance of the patient-level model also enhanced the measures’ accuracy.

### Limitations

There are several potential limitations to consider. We restricted our analysis to claims using *ICD-9-CM* codes because of our interest in using a large sample and not mixing *ICD-9-CM* and *ICD-10-CM* codes. Our approach would need to be thoroughly tested with *ICD-10-CM* codes, although, from preliminary experiments, we do not expect the results to change substantially, even with a potentially greater impact from the 0.5% frequency restriction. With the greater specificity of *ICD-10-CM* codes, performance may improve further. Another potential issue is that our approach could ignore low-frequency codes; however, they do not exert much influence in groupers even when included. Additionally, using least absolute shrinkage and selection operator analysis for code selection could result in some relevant codes being dropped owing to correlation, possibly diminishing predictive power. We chose this method owing to its ease in selecting a specified number of codes as required by our hospital-level algorithm and to avoid possible issues that multicollinearity can cause in HGLM.^22^ Nevertheless, we were consistent in our approach in all of the new models, so it should not have biased our comparative assessment. Also, we chose not to modify the currently used hospital-level HGLM method. However, a 2-stage HGLM or other novel methods would have allowed us to consider more sophisticated machine learning methods for patient-level risk adjustment.^23,24^ In addition, our approach may not be applicable for data with extreme outcome rates in combination with extreme risk factor prevalence in which using logistic regression would be problematic owing to the complete or quasi-complete separation. Moreover, the large number of variables included in the final models reduce the computational efficiency. Also, to our knowledge, there are few contemporary studies on the accuracy of POA codes, and the studies that have been published provide conflicting results on their accuracy.^25,26,27^ As such, there is a need for more research on this topic. Additionally, the use of individual diagnostic codes could inadvertently augment the effect of clinically insignificant differences in code use among hospitals. To address this possibility, it might be necessary to monitor the coding and potentially reselect codes and refit the model at more frequent intervals.

## Conclusions

We identify several key strategies, made possible in part by an evolution of claims coding, to improve prediction models with implications for research and performance measurement. We found that incorporating POA coding into risk variable definitions, distinguishing between diagnoses coded in the claims within 12 months before the index admission from those coded at admission, and disaggregating from groupers into individual *ICD-9-CM* codes was associated with substantially improved patient-level and hospital-level models for AMI, HF, and pneumonia mortality. Disaggregating the groupers appeared to provide the largest improvement in many of the risk models. It also enabled hospital performance measures to identify more hospitals as better or worse than the national mean for all 3 conditions. These findings suggest that there may be opportunities to improve the risk models for research and outcome performance measures. In particular, further investigation of the effect of such changes on measure score validity might provide additional support for incorporating these methods in the future.

## Notes

Supplement. **eMethods.** Present on Admission–Exempt *International Classification of Diseases, Ninth Revision, Clinical Modification* Code Methodology **eFigure 1.** Flowchart to Determine Which Individual *ICD-9-CM* Codes Were Selected for the Individual-Codes Logistic Regression Model **eFigure 2.** Comparison of Receiver Operating Characteristic Curves **eFigure 3.** Kernel Density Plots Comparing the Log Odds of the CMS and Individual-Codes Patient-Level 30-Day Mortality Models **eFigure 4.** Comparison of Distribution of Hospital Risk-Standardized Mortality Rates for CMS vs Individual-Codes Hospital-Level 30-Day Mortality Models **eTable 1.** Shift Tables Comparing the Predicted Risk of the CMS and Individual-Codes Models **eTable 2.** Top 50 *ICD-9-CM* Codes Selected by the Individual-Codes Model for Acute Myocardial Infarction Compared With Their Corresponding Version 22 HCC Codes **eTable 3.** Top 50 *ICD-9-CM* Codes Selected by the Individual-Codes Model for Heart Failure Compared With Their Corresponding Version 22 HCC Codes **eTable 4.** Top 50 *ICD-9-CM* Codes Selected by the Individual-Codes Model for Pneumonia Compared With Their Corresponding Version 22 HCC Codes **eTable 5.** Centers for Medicare & Medicaid Services Publicly Reported Performance Categories for the CMS Model Compared With the Individual-Codes Model for 30-Day Mortality Measures Among Hospitals With at Least 25 Cases

## References

[1] Krumholz HM, Normand SL, Spertus JA, Shahian DM, Bradley EH Measuring performance for treating heart attacks and heart failure: the case for outcomes measurement. Health Aff (Millwood). 2007;26(1):-. doi:10.1377/hlthaff.26.1.75 17211016

[2] Bratzler DW, Normand SL, Wang Y, An administrative claims model for profiling hospital 30-day mortality rates for pneumonia patients. PLoS One. 2011;6(4):e17401. doi:10.1371/journal.pone.0017401 21532758 PMC3075250

[3] Keenan PS, Normand SL, Lin Z, An administrative claims measure suitable for profiling hospital performance on the basis of 30-day all-cause readmission rates among patients with heart failure. Circ Cardiovasc Qual Outcomes. 2008;1(1):29-37. doi:10.1161/CIRCOUTCOMES.108.802686 20031785

[4] Krumholz HM, Lin Z, Drye EE, An administrative claims measure suitable for profiling hospital performance based on 30-day all-cause readmission rates among patients with acute myocardial infarction. Circ Cardiovasc Qual Outcomes. 2011;4(2):243-252. doi:10.1161/CIRCOUTCOMES.110.957498 21406673 PMC3350811

[5] Krumholz HM, Wang Y, Mattera JA, An administrative claims model suitable for profiling hospital performance based on 30-day mortality rates among patients with an acute myocardial infarction. Circulation. 2006;113(13):1683-1692. doi:10.1161/CIRCULATIONAHA.105.611186 16549637

[6] Krumholz HM, Wang Y, Mattera JA, An administrative claims model suitable for profiling hospital performance based on 30-day mortality rates among patients with heart failure. Circulation. 2006;113(13):1693-1701. doi:10.1161/CIRCULATIONAHA.105.611194 16549636

[7] Lindenauer PK, Normand SL, Drye EE, Development, validation, and results of a measure of 30-day readmission following hospitalization for pneumonia. J Hosp Med. 2011;6(3):142-150. doi:10.1002/jhm.890 21387551

[8] Medicare Learning Network Present on admission (POA) indicator editing for Maryland waiver hospitals. https://www.cms.gov/Outreach-and-Education/Medicare-Learning-Network-MLN/MLNMattersArticles/downloads/MM8709.pdf. Accessed May 23, 2019.

[9] Pope GC, Ellis RP, Ash AS, Diagnostic cost group hierarchical condition category models for Medicare risk adjustment: final report. https://www.cms.gov/Research-Statistics-Data-and-Systems/Statistics-Trends-and-Reports/Reports/downloads/pope_2000_2.pdf. Accessed June 6, 2019.

[10] Pope GC, Kautter J, Ellis RP, Risk adjustment of Medicare capitation payments using the CMS-HCC model. Health Care Financ Rev. 2004;25(4):119-141. 15493448 PMC4194896

[11] Spertus JV, T Normand SL, Wolf R, Cioffi M, Lovett A, Rose S Assessing hospital performance following percutaneous coronary intervention using big data. Circ Cardiovasc Qual Outcomes. 2016;9(6):659-669. doi:10.1161/CIRCOUTCOMES.116.002826 28263941 PMC5341139

[12] Lindenauer PK, Bernheim SM, Grady JN, The performance of US hospitals as reflected in risk-standardized 30-day mortality and readmission rates for Medicare beneficiaries with pneumonia. J Hosp Med. 2010;5(6):E12-E18. doi:10.1002/jhm.822 20665626

[13] World Health Organization International Classification of Diseases, Ninth Revision, Clinical Modification (ICD-9-CM). Geneva, Switzerland: World Health Organization; 1977.

[14] Yale New Haven Health Services Corporation; Center for Outcomes Research and Evaluation Archived resources: mortality measures. http://www.qualitynet.org/dcs/ContentServer?c=Page&pagename=QnetPublic%2FPage%2FQnetTier3&cid=1228774398696. Accessed May 23, 2019.

[15] DeLong ER, DeLong DM, Clarke-Pearson DL Comparing the areas under two or more correlated receiver operating characteristic curves: a nonparametric approach. Biometrics. 1988;44(3):837-845. doi:10.2307/2531595 3203132

[16] Daniels MJ, Gatsonis C Hierarchical generalized linear models in the analysis of variations in health care utilization. J Am Stat Assoc. 1999;94(445):29-42. doi:10.1080/01621459.1999.10473816

[17] Normand ST, Shahian DM Statistical and clinical aspects of hospital outcomes profiling. Stat Sci. 2007;22(2):206-226. doi:10.1214/088342307000000096

[18] Bin Abdullah M On a robust correlation coefficient. J Royal Stat Soc, Series D (The Statistician). 1990;39(4):455-460.

[19] Wicklin R How to understand weight variables in statistical analyses. https://blogs.sas.com/content/iml/2017/10/02/weight-variables-in-statistics-sas.html. Accessed May 23, 2019.

[20] US Centers for Medicare & Medicaid Services Hospital Compare. https://www.medicare.gov/hospitalcompare/search.html. Accessed May 23, 2019.

[21] COPSS-CMS White Paper Committee Statistical issues in assessing hospital performance. https://www.cms.gov/Medicare/Quality-Initiatives-Patient-Assessment-Instruments/HospitalQualityInits/Downloads/Statistical-Issues-in-Assessing-Hospital-Performance.pdf. Accessed May 23, 2019.

[22] Yu H, Jiang S, Land KC Multicollinearity in hierarchical linear models. Soc Sci Res. 2015;53:118-136. doi:10.1016/j.ssresearch.2015.04.008 26188442

[23] Rose S Mortality risk score prediction in an elderly population using machine learning. Am J Epidemiol. 2013;177(5):443-452. doi:10.1093/aje/kws241 23364879

[24] Kunz LM, Rose S, Spiegelman D, Normand ST An overview of statistical approaches for comparative effectiveness research In: Gatsonis C, Morton SC, eds. Methods in Comparative Effectiveness Research. Boca Raton, FL: Chapman and Hall/CRC; 2017:23-58.

[25] Cram P, Bozic KJ, Callaghan JJ, Lu X, Li Y Use of present-on-admission indicators for complications after total knee arthroplasty: an analysis of Medicare administrative data. J Arthroplasty. 2014;29(5):923-928.e2. doi:10.1016/j.arth.2013.11.002 24530205 PMC4451935

[26] Khanna RR, Kim SB, Jenkins I, Predictive value of the present-on-admission indicator for hospital-acquired venous thromboembolism. Med Care. 2015;53(4):e31-e36. 23552437 10.1097/MLR.0b013e318286e34f

[27] Squitieri L, Waxman DA, Mangione CM, Evaluation of the present-on-admission indicator among hospitalized fee-for-service Medicare patients with a pressure ulcer diagnosis: coding patterns and impact on hospital-acquired pressure ulcer rates. Health Serv Res. 2018;53(suppl 1):2970-2987. doi:10.1111/1475-6773.12822 29552746 PMC6056601

