# Supplementary material for: Comparative Effectiveness of New Approaches to Improve Mortality Risk Models From Medicare Claims Data
Source: JAMA Netw Open. 2019 Jul 17;2(7):e197314. doi: 10.1001/jamanetworkopen.2019.7314 (PMC6647547; doi:10.1001/jamanetworkopen.2019.7314)
Supplement: Supplement. — eMethods. Present on Admission–Exempt International Classification of Diseases, Ninth Revision, Clinical Modification Code Methodology eFigure 1. Flowchart to Determine Which Individual ICD-9-CM Codes Were Selected for the Individual-Codes Logistic Regression Model eFigure 2. Comparison of Receiver Operating Characteristic Curves eFigure 3. Kernel Density Plots Comparing the Log Odds of the CMS and Individual-Codes Patient-Level 30-Day Mortality Models eFigure 4. Comparison of Distribution of Hospital Risk-Standardized Mortality Rates for CMS vs Individual-Codes Hospital-Level 30-Day Mortality Models eTable 1. Shift Tables Comparing the Predicted Risk of the CMS and Individual-Codes Models eTable 2. Top 50 ICD-9-CM Codes Selected by the Individual-Codes Model for Acute Myocardial Infarction Compared With Their Corresponding Version 22 HCC Codes eTable 3. Top 50 ICD-9-CM Codes Selected by the Individual-Codes Model for Heart Failure Compared With Their Corresponding Version 22 HCC Codes eTable 4. Top 50 ICD-9-CM Codes Selected by the Individual-Codes Model for Pneumonia Compared With Their Corresponding Version 22 HCC Codes eTable 5. Centers for Medicare & Medicaid Services Publicly Reported Performance Categories for the CMS Model Compared With the Individual-Codes Model for 30-Day Mortality Measures Among Hospitals With at Least 25 Cases [file jamanetwopen-2-e197314-s001.pdf]

Supplementary Online Content

Krumholz HM, Coppi AC, Warner F, et al. Comparative effectiveness of new approaches to improve mortality risk models from Medicare claims data. *JAMA Netw Open*. 2019;2(7):e197314. doi:0.1001/jamanetworkopen.2019.7314

eMethods. Present on Admission–Exempt *International Classification of Diseases, Ninth Revision, Clinical Modification* Code Methodology

eFigure 1. Flowchart to Determine Which Individual *ICD-9-CM* Codes Were Selected for the Individual-Codes Logistic Regression Model

eFigure 2. Comparison of Receiver Operating Characteristic Curves

eFigure 3. Kernel Density Plots Comparing the Log Odds of the CMS and Individual-Codes Patient-Level 30-Day Mortality Models

eFigure 4. Comparison of Distribution of Hospital Risk-Standardized Mortality Rates for CMS vs Individual-Codes Hospital-Level 30-Day Mortality Models

eTable 1. Shift Tables Comparing the Predicted Risk of the CMS and Individual-Codes Models

eTable 2. Top 50 *ICD-9-CM* Codes Selected by the Individual-Codes Model for Acute Myocardial Infarction Compared With Their Corresponding Version 22 HCC Codes

eTable 3. Top 50 *ICD-9-CM* Codes Selected by the Individual-Codes Model for Heart Failure Compared With Their Corresponding Version 22 HCC Codes

eTable 4. Top 50 *ICD-9-CM* Codes Selected by the Individual-Codes Model for Pneumonia Compared With Their Corresponding Version 22 HCC Codes

eTable 5. Centers for Medicare & Medicaid Services Publicly Reported Performance Categories for the CMS Model Compared With the Individual-Codes Model for 30-Day Mortality Measures Among Hospitals With at Least 25 Cases

This supplementary material has been provided by the authors to give readers additional information about their work.

© 2019 Krumholz HM et al. *JAMA Network Open.*

eMethods. Present on Admission–Exempt International Statistical Classification of Diseases, Ninth Revision, Clinical Modification Code Methodology

As part of a currently unpublished separate project, we convened a technical working group of clinical experts who provided clinical rationale for categorizing the POA-exempt codes as “always POA” or “don’t count as POA” based on their clinical expertise, the rationale from the official ICD-9-CM coding guidelines, and our recommendations. The majority of the ICD-9-CM codes included in the POA-exempt list were subsequent, sequela, or congenital codes. Based on ICD-9-CM coding guidelines, we coded these types of codes as “always POA” because, by definition, subsequent and sequela codes should not be used for conditions acquired during a hospitalization in which the patient is receiving active treatment for that condition. Additional groups of codes were further reviewed by clinical experts to determine whether they should be counted as “always POA” or not. We excluded POA-exempt codes from the “always POA” list based on any one of 4 criteria: 1) they were not relevant to the Medicare population, such as codes related to pregnancy, childbirth, and children’s health statuses; 2) they could potentially be coded as a complication of care during an index admission or POA, such as exposure to toxic substances or medication overdoses; 3) they provided no relevant information about a patient’s health status or reason for admission, such as family history codes or encounter codes, which indicate that a patient has an encounter for a procedure but does not specify that the procedure was performed; or 4) they were not mandatory for reporting. The latter exclusion pertained specifically to S00-T88 injury, poisoning, and certain other consequences of external causes and V00-Y99 external causes of morbidity, which are claims collected for the purposes of injury research.

The lists of ICD-9-CM POA-exempt codes resulting from the above were used as the basis for determining which of a patient’s diagnosis codes neither flagged POA=Y nor POA=N would be allowable data for the individual codes model fit.

The project was conducted by the Yale New Haven Health Services Corporation–Center for Outcomes Research and Evaluation (CORE). The research was led by the following individuals affiliated with CORE:

Elizabeth W. Triche, PhD

Shengfan Zhou, MS

Danielle Purvis, MPH

Grace Glennon, MS

Kofi Dwamena, BS

eFigure 1. Flowchart to Determine Which Individual ICD-9-CM Codes Were Selected for the Individual-Codes Logistic Regression Model

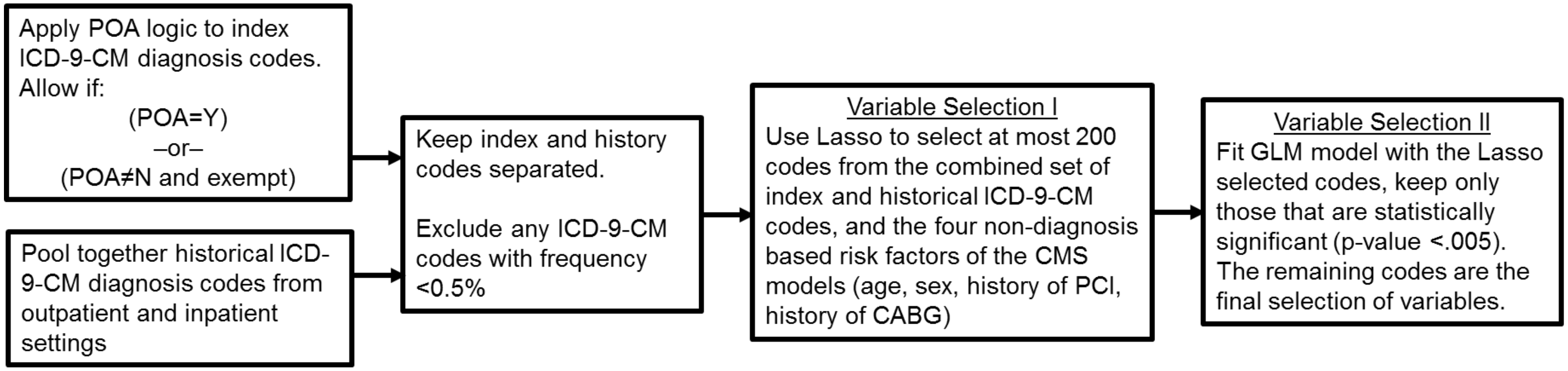

CABG, Coronary Artery Bypass Grafting; CMS, Centers for Medicare & Medicaid Services; GLM, Generalized Linear Models; ICD-9-CM, International Classification of Diseases, Ninth Revision, Clinical Modification; PCI, Percutaneous Coronary Intervention; Lasso, Least Absolute Shrinkage and Selection Operator; POA, Present on Admission

© 2019 Krumholz HM et al. *JAMA Network Open.*

eFigure 2. Comparison of Receiver Operating Characteristic Curves

A. Receiver Operating Characteristic curves

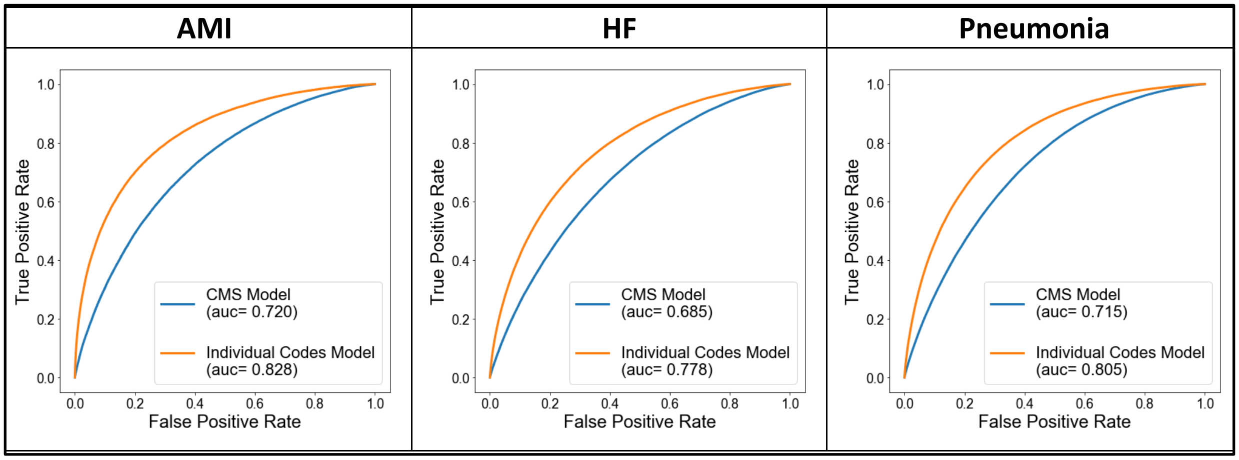

B. Calibration plots

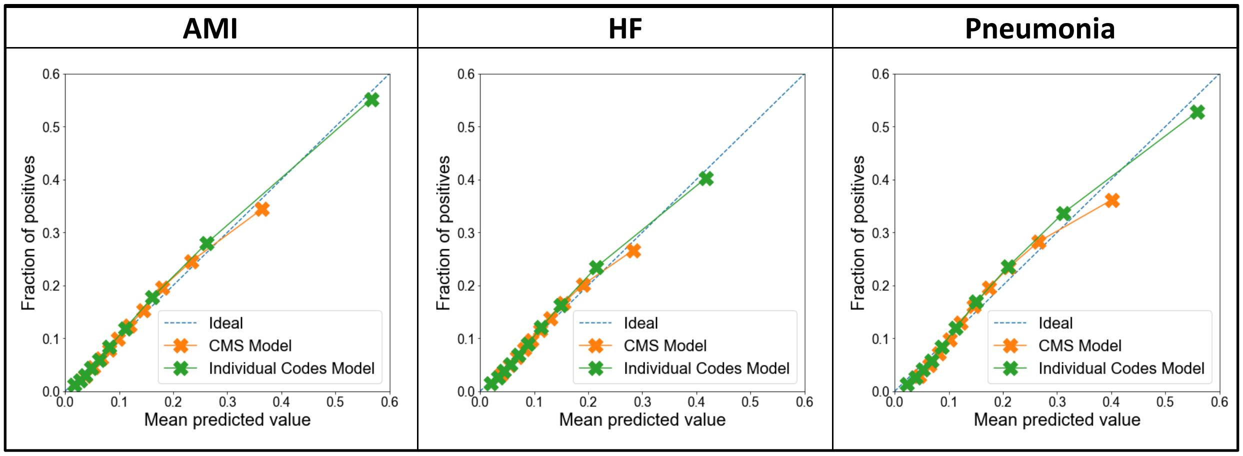

AMI, Acute Myocardial Infarction; AUC, area under the curve; CMS, Centers for Medicare & Medicaid Services; HF, Heart Failure

© 2019 Krumholz HM et al. *JAMA Network Open.*

eFigure 3. Kernel Density Plots Comparing the Log Odds of the CMS and Individual-Codes Patient-Level 30-Day Mortality Models

A. Acute myocardial infarction

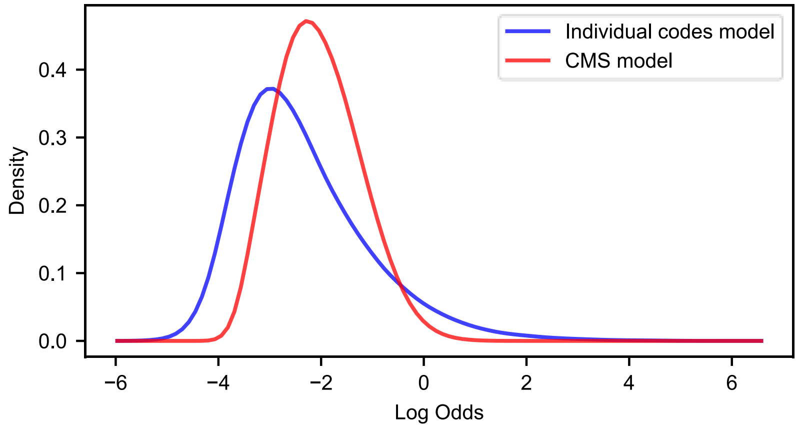

B. Heart failure

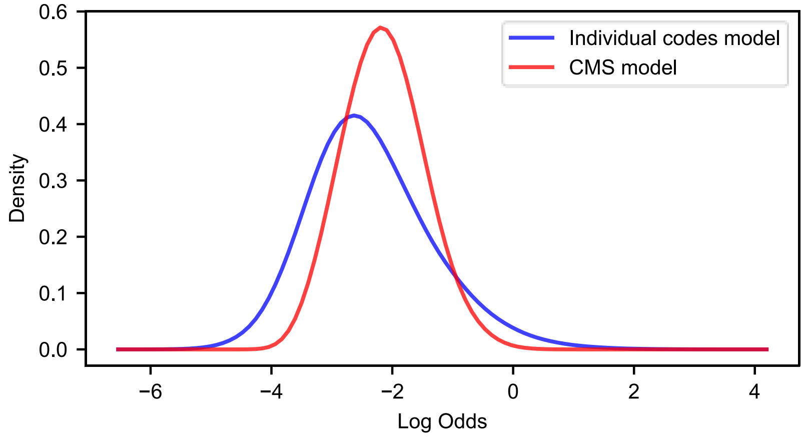

C. Pneumonia

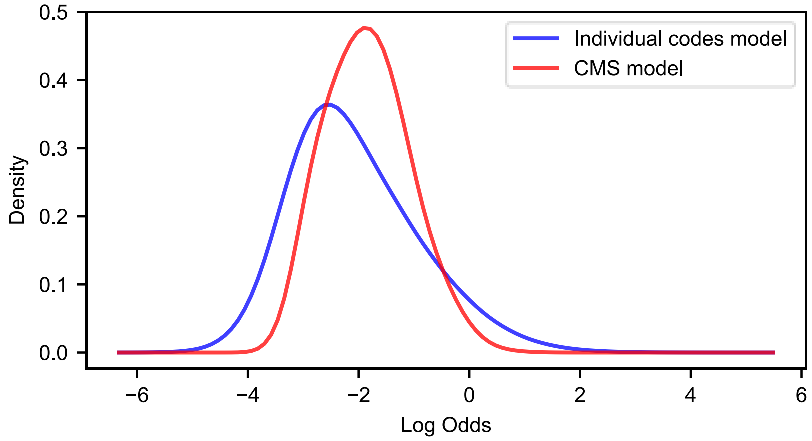

More spread-out distribution associated with better discrimination of patient-level risk.
CMS, Centers for Medicare & Medicaid Services

© 2019 Krumholz HM et al. *JAMA Network Open.*

eFigure 4. Comparison of Distribution of Hospital Risk-Standardized Mortality Rates for CMS vs Individual-Codes Hospital-Level 30-Day Mortality Models

A. Acute myocardial infarction

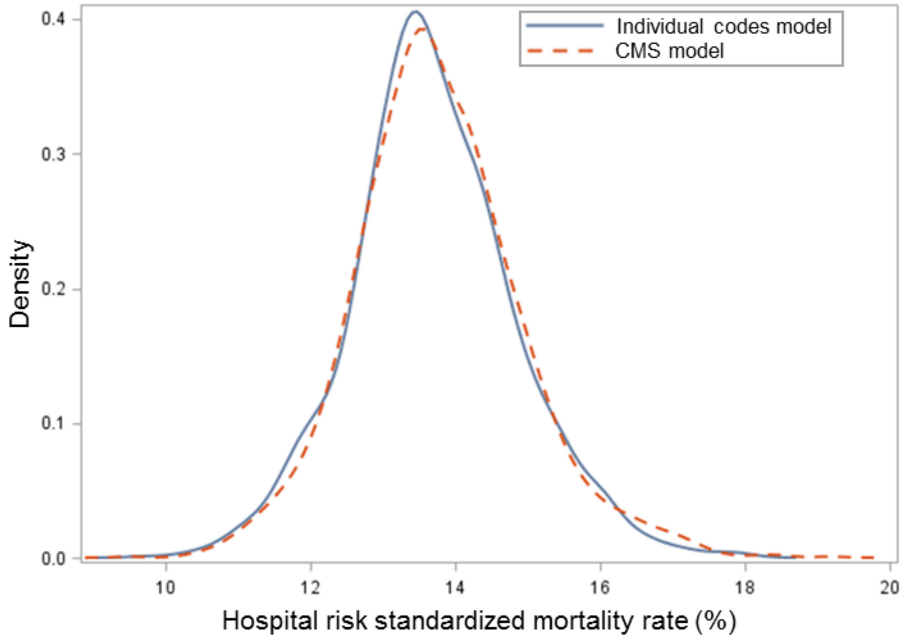

B. Heart failure

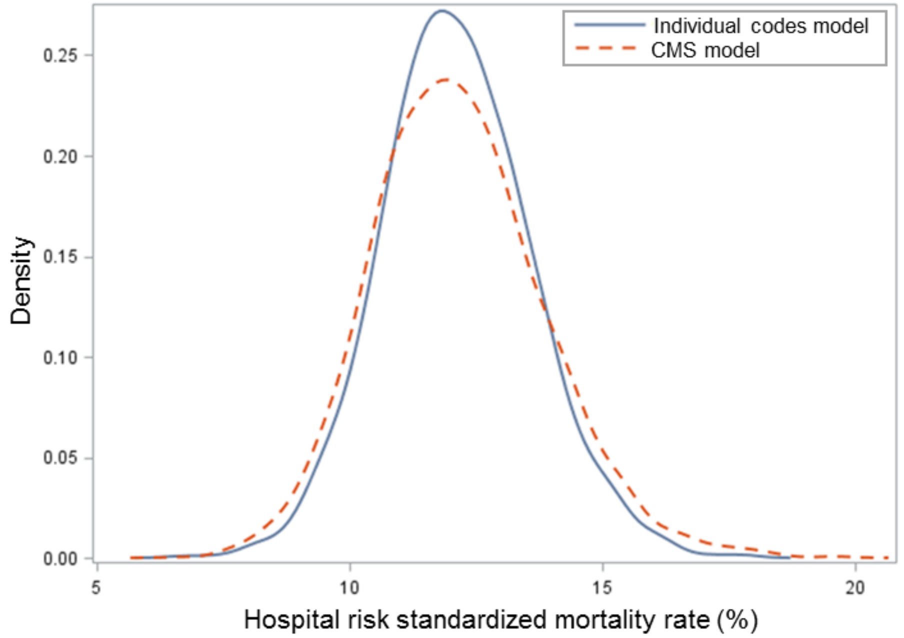

eFigure 4. (continued)

© 2019 Krumholz HM et al. *JAMA Network Open.*

C. Pneumonia

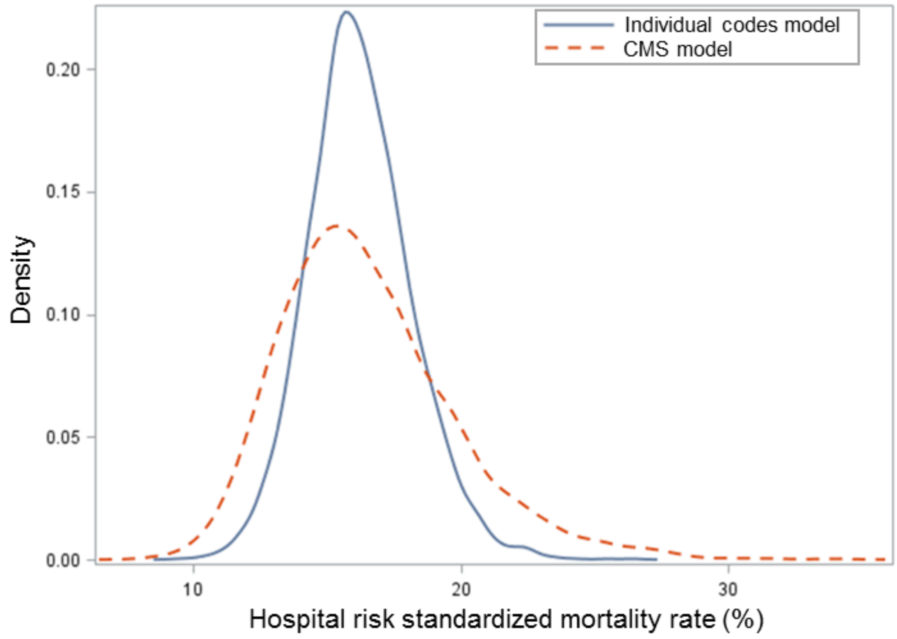

More spread-out distribution associated with better discrimination of hospital performance.
CMS, Centers for Medicare & Medicaid Services

© 2019 Krumholz HM et al. *JAMA Network Open.*

eTable 1. Shift Tables Comparing the Predicted Risk of the CMS And Individual-Codes Models
(Each cell in the table contains the number of index admissions in the intersection of the 2 models' predicted risk categories [row vs col], and below it, within square brackets, is the observed outcome rate for that subset of index admissions.)

A. Acute myocardial infarction

Acute myocardial infarction		CMS model predicted risk					Row totals
		<5%	5%-10%	10%-25%	25%-50%	>50%	
Individual-codes model predicted risk	<5%	41205 [1.6%]	67502 [2.5%]	22191 [3.9%]	693 [8.2%]	3 [33.3%]	131594 [2.5%]
	5%–10%	4178 [6.6%]	33250 [5.9%]	50627 [7.5%]	4248 [10.2%]	59 [6.8%]	92362 [7.0%]
	10%–25%	1254 [18.2%]	12945 [15.9%]	50511 [16.3%]	16165 [19.9%]	552 [25.0%]	81427 [17.0%]
	25%–50%	379 [37.7%]	3015 [37.9%]	18471 [36.0%]	13085 [36.3%]	1088 [38.1%]	36038 [36.4%]
	>50%	318 [66.4%]	2104 [68.9%]	8171 [67.7%]	7901 [63.4%]	1260 [62.5%]	19754 [65.8%]
Column totals		47334 [3.2%]	118816 [7.0%]	149971 [16.7%]	42092 [32.0%]	2962 [45.4%]	361175 [13.8%]

B. Heart failure

Heart failure		CMS model predicted risk					Row totals
		<5%	5%-10%	10%-25%	25%-50%	>50%	
Individual-codes model predicted risk	<5%	63192 [1.8%]	109590 [2.6%]	38428 [3.9%]	789 [6.2%]	1 [0.0%]	212000 [2.6%]
	5%–10%	14107 [5.7%]	98802 [6.2%]	103270 [7.7%]	4673 [10.5%]	16 [12.5%]	220868 [7.0%]
	10%–25%	3244 [15.4%]	46807 [15.1%]	133985 [17.0%]	17696 [19.7%]	156 [23.1%]	201888 [16.8%]
	25%–50%	350 [36.3%]	6519 [36.0%]	44602 [34.5%]	14169 [34.7%]	264 [32.6%]	65904 [34.7%]
	>50%	45 [60.0%]	851 [56.5%]	8872 [55.7%]	6147 [54.2%]	215 [56.3%]	16130 [55.2%]
Column totals		80938 [3.2%]	262569 [7.2%]	329157 [16.0%]	43474 [28.3%]	652 [37.6%]	716790 [12.1%]

© 2019 Krumholz HM et al. *JAMA Network Open.*

eTable 1. (continued)

C. Pneumonia

Pneumonia		CMS model predicted risk					Row totals
		<5%	5%-10%	10%-25%	25%-50%	>50%	
Individual-codes model predicted risk	<5%	52893 [1.2%]	119951 [1.9%]	55358 [3.7%]	2941 [5.5%]	35 [2.9%]	231178 [2.2%]
	5%–10%	12042 [4.7%]	97217 [5.0%]	142004 [7.2%]	16082 [10.3%]	374 [15.0%]	267719 [6.5%]
	10%–25%	3169 [13.5%]	46114 [14.3%]	184141 [17.4%]	55994 [21.1%]	2505 [24.4%]	291923 [17.6%]
	25%–50%	330 [38.5%]	7548 [35.7%]	76849 [36.7%]	51382 [37.7%]	4186 [39.5%]	140295 [37.1%]
	>50%	19 [52.6%]	824 [59.7%]	20888 [57.9%]	30382 [58.5%]	4997 [60.1%]	57110 [58.4%]
Column totals		68453 [2.6%]	271654 [6.2%]	479240 [17.6%]	156781 [32.4%]	12097 [44.0%]	988225 [16.1%]

These tables show the individual-codes model more accurately reclassifying risk categories based on the CMS model.
CMS, Centers for Medicare & Medicaid Services

© 2019 Krumholz HM et al. *JAMA Network Open.*

eTable 2. Top 50 *ICD-9-CM* Codes Selected by the Individual-Codes Model For Acute Myocardial Infarction Compared With Their Corresponding Version 22 HCC Codes

**Acute myocardial infarction** [Full cohort mortality rate: 13.8%]							
**ICD-9-CM**				**v22 HCC**			
**Code**	**Description**	**Frequency of occurrence**	**Mortality rate**	**Code**	**Description**	**Frequency of occurrence**	**Mortality rate**
3481	Anoxic brain damage	1.0%	75.1%	80	Coma, brain compression/anoxic damage	1.2%	73.8%
78551	Cardiogenic shock	4.5%	51.4%	84	Cardio-respiratory failure and shock	16.6%	33.5%
4275	Cardiac arrest	2.2%	59.7%	84	Cardio-respiratory failure and shock	16.6%	33.5%
V4986	Do not resuscitate status	10.3%	37.2%	179	Minor symptoms, signs, findings	25.8%	22.4%
42983	Takotsubo syndrome	1.2%	6.1%	98	Other and unspecified heart disease	4.9%	10.2%
4010	Malignant hypertension	1.5%	5.5%	95	Hypertension	52.7%	10.6%
5845	Acute kidney failure with lesion of tubular necrosis	1.3%	43.6%	135	Acute renal failure	16.5%	27.0%
7837	Failure to thrive-adult	1.0%	40.6%	178	Major symptoms, abnormalities	24.7%	14.5%
1629	Malignant neoplasm of bronchus and lung, unspecified	0.6%	34.1%	9	Lung and other severe cancers	1.5%	29.7%
43491	Cerebral artery occlusion, unspecified with cerebral infarction	0.5%	31.1%	100	Ischemic or unspecified stroke	0.7%	29.3%
40300	Cerebral artery occlusion, unspecified with cerebral infarction	0.8%	7.9%	139	Chronic kidney disease, mild or unspecified (Stages 1-2 or unspecified)	24.0%	16.9%
4660	Acute bronchitis	0.6%	8.5%	118	Other respiratory disorders	9.6%	10.6%
7804	Dizziness and giddiness	0.6%	6.8%	179	Minor symptoms, signs, findings	25.8%	22.4%
7994	Cachexia	0.7%	37.1%	21	Protein-calorie malnutrition	3.4%	30.7%
41519	Other pulmonary embolism and infarction	0.5%	26.2%	107	Vascular disease with complications	1.1%	26.2%

© 2019 Krumholz HM et al. *JAMA Network Open.*

eTable 2. (Continued)

ICD-9-CM				v22 HCC			
Code	Description	Frequency of occurrence	Mortality rate	Code	Description	Frequency of occurrence	Mortality rate
2762	Acidosis	4.5%	41.0%	24	Disorders of fluid/electrolyte/acid-base balance	21.8%	23.4%
Hx_1985	Secondary malignant neoplasm of bone and bone marrow	0.6%	28.4%	8	Metastatic cancer and acute leukemia	1.7%	26.8%
41071	Subendocardial infarction, initial episode of care	75.5%	12.0%	86	Acute myocardial infarction	0.6%	24.8%
40290	Unspecified hypertensive heart disease without heart failure	0.8%	6.2%	94	Hypertensive heart disease	0.9%	5.9%
51884	Acute and chronic respiratory fail	1.6%	27.7%	84	Cardio-respiratory failure and shock	16.6%	33.5%
51881	Acute respiratory failure	8.2%	37.5%	84	Cardio-respiratory failure and shock	16.6%	33.5%
41091	Acute myocardial infarction of unspecified site, initial episode of care	4.3%	33.4%	86	Acute myocardial infarction	0.6%	24.8%
570	Acute necrosis of liver	0.7%	59.2%	30	Acute liver failure/disease	0.8%	57.7%
42831	Acute diastolic heart failure	1.7%	12.6%	85	Congestive heart failure	44.6%	18.7%
04149	Other and unspecified Escherichia coli (E. coli)	1.5%	13.6%	7	Other infectious diseases	4.4%	15.2%
0389	Unspecified septicemia	0.8%	39.9%	2	Septicemia, sepsis, systemic inflammatory response syndrome/shock	1.7%	34.5%
42832	Chronic diastolic heart failure	2.5%	13.3%	85	Congestive heart failure	44.6%	18.7%
4270	Paroxysmal supraventricular tachycardia	0.6%	9.8%	96	Specified heart arrhythmias	28.6%	18.0%
5849	Acute kidney failure, unspecified	15.3%	25.6%	135	Acute renal failure	16.5%	27.0%

© 2019 Krumholz HM et al. *JAMA Network Open.*

eTable 2. (Continued)

ICD-9-CM				v22 HCC			
Code	Description	Frequency of occurrence	Mortality rate	Code	Description	Frequency of occurrence	Mortality rate
5856	End stage renal disease	4.1%	21.1%	136	Chronic kidney disease, Stage 5	4.5%	21.4%
Hx_1991	Other malignant neoplasm without specification of site	0.6%	27.3%	12	Breast, prostate, and other cancers and tumors	8.0%	14.6%
42830	Diastolic heart failure, unspecified	1.1%	14.5%	85	Congestive heart failure	44.6%	18.7%
Hx_23875	Myelodysplastic syndrome, unspecified	0.6%	25.1%	46	Severe hematological disorders	1.0%	23.2%
49322	Chronic obstructive asthma with (acute) exacerbation	0.6%	11.1%	111	Chronic obstructive pulmonary disease	21.2%	15.6%
79311	Solitary pulmonary nodule	0.7%	7.8%	179	Minor symptoms, signs, findings	25.8%	22.4%
4589	Hypotension, unspecified	3.8%	24.6%	109	Other circulatory disease	7.4%	20.2%
262	Other severe protein-calorie malnutrition	0.6%	34.6%	21	Protein-calorie malnutrition	3.4%	30.7%
7812	Abnormality of gait	0.9%	12.5%	178	Major symptoms, abnormalities	24.7%	14.5%
514	Pulmonary congestion and hypostasis	0.7%	29.8%	116	Viral and unspecified pneumonia, pleurisy	7.8%	25.4%
5852	Chronic kidney disease, Stage II (mild)	1.2%	11.0%	139	Chronic kidney disease, mild or unspecified (Stages 1-2 or unspecified)	24.0%	16.9%
42781	Sinoatrial node dysfunction	1.5%	11.9%	96	Specified heart arrhythmias	28.6%	18.0%
70703	Pressure ulcer, lower back	0.9%	33.8%	160	Pressure pre-ulcer skin changes or unspecified stage	1.4%	31.4%
42611	First degree atrioventricular block	1.7%	10.0%	97	Other heart rhythm and conduction disorders	16.1%	14.5%
2760	Hyperosmolality and/or hypernatremia	1.0%	38.1%	24	Disorders of fluid/electrolyte/acid-base balance	21.8%	23.4%

© 2019 Krumholz HM et al. *JAMA Network Open.*

eTable 2. (Continued)

ICD-9-CM				v22 HCC			
**Code**	**Description**	**Frequency of occurrence**	**Mortality rate**	**Code**	**Description**	**Frequency of occurrence**	**Mortality rate**
49122	Obstructive chronic bronchitis with acute bronchitis	0.7%	10.7%	111	Chronic obstructive pulmonary disease	21.2%	15.6%
2752	Disorders of magnesium metabolism	3.1%	11.3%	26	Other endocrine/metabolic/nutritional disorders	24.4%	14.1%
42833	Acute on chronic diastolic heart failure	3.7%	16.2%	85	Congestive heart failure	44.6%	18.7%
4280	Congestive heart failure, unspecified	37.4%	19.3%	85	Congestive heart failure	44.6%	18.7%
4239	Unspecified disease of pericardium	0.5%	20.7%	90	Heart infection/inflammation, except rheumatic	0.7%	21.7%
41001	Acute myocardial infarction of anterolateral wall, initial episode of care	1.8%	21.8%	86	Acute myocardial infarction	0.6%	24.8%

The above ranked list contains only the individual ICD-9-CM codes that were chosen by the variable selection method. Of the non-diagnosis-based variables that were included for consideration (age, sex, history of percutaneous coronary intervention procedure, and history of coronary artery bypass grafting procedure), the method selected only age.

A prepended "Hx_" on the ICD-9-CM or on the mapped v22 HCC code label indicates that it occurred during the 12-prior claim history; otherwise, the code is from the index admission.

ICD-9-CM, International Classification of Diseases, Ninth Revision, Clinical Modification; v22 HCC, Version 22 Hierarchical Condition Categories

eTable 3. Top 50 *ICD-9-CM* Codes Selected by the Individual-Codes Model for Heart Failure Compared With Their Corresponding Version 22 HCC Codes

**Heart failure**<br>[Full Cohort Mortality Rate: 12.1%]							
**ICD-9-CM**				**v22 HCC**			
**Code**	**Description**	**Frequency of occurrence**	**Mortality rate**	**Code**	**Description**	**Frequency of occurrence**	**Mortality rate**
78551	Cardiogenic shock	0.6%	47.5%	84	Cardio-respiratory failure and shock	29.4%	16.6%
V4986	Do not resuscitate status	13.5%	27.4%	179	Minor symptoms, signs, findings	33.5%	17.6%
1629	Malignant neoplasm of bronchus and lung, unspecified	0.5%	27.7%	9	Lung and other severe cancers	1.7%	22.6%
7837	Adult failure to thrive	1.8%	35.5%	178	Major symptoms, abnormalities	31.4%	13.4%
5845	Acute kidney failure with lesion of tubular necrosis	0.9%	27.9%	135	Acute renal failure	21.9%	18.5%
4010	Malignant hypertension	1.2%	5.0%	95	Hypertension	37.4%	9.2%
5070	Pneumonitis due to inhalation of food or vomitus	0.8%	34.1%	114	Aspiration and specified bacterial pneumonias	1.0%	31.4%
78659	Other chest pain	1.2%	4.6%	178	Major symptoms, abnormalities	31.4%	13.4%
262	Other severe protein-calorie malnutrition	1.0%	32.3%	21	Protein-calorie malnutrition	5.2%	27.4%
4659	Acute upper respiratory infections of unspecified site	0.5%	6.0%	131	Other ear, nose, throat, and mouth disorders	2.60%	9.10%
7994	Cachexia	1.0%	32.5%	21	Protein-calorie malnutrition	5.2%	27.4%
4589	Hypotension, unspecified	3.2%	24.7%	109	Other circulatory disease	9.9%	16.3%
34831	Metabolic encephalopathy	1.2%	29.6%	50	Delirium and encephalopathy	3.5%	27.8%
4660	Acute bronchitis	1.4%	6.5%	118	Other respiratory disorders	18.4%	9.4%
51884	Acute and chronic respiratory failure	6.6%	19.9%	84	Cardio-respiratory failure and shock	29.4%	16.6%
34830	Encephalopathy, unspecified	1.1%	28.8%	50	Delirium and encephalopathy	3.5%	27.8%

© 2019 Krumholz HM et al. *JAMA Network Open.*

eTable 3. (Continued)

ICD-9-CM				v22 HCC			
**Code**	**Description**	**Frequency of occurrence**	**Mortality rate**	**Code**	**Description**	**Frequency of occurrence**	**Mortality rate**
78097	Altered mental status	0.8%	25.3%	179	Minor symptoms, signs, findings	33.5%	17.6%
2760	Hyperosmolality	1.2%	28.8%	24	Disorders of fluid/electrolyte/acid-base balance	28.6%	16.5%
40401	Hypertensive heart and chronic kidney disease, malignant, with heart failure and with chronic kidney disease stage I through stage IV, or unspecified	0.6%	6.2%	85	Congestive heart failure	87.0%	11.8%
V1581	Personal history of noncompliance with medical treatment, presenting hazards to health	6.0%	6.3%	196	History of disease	54.2%	10.8%
40300	Hypertensive chronic kidney disease, malignant, with chronic kidney disease stage I through stage IV, or unspecified	1.1%	7.0%	139	Chronic kidney disease, mild or unspecified (Stages 1-2 or unspecified)	35.7%	13.5%
41071	Subendocardial infarction, initial episode of care	2.3%	21.2%	86	Acute myocardial infarction	2.6%	21.9%
2639	Unspecified protein-calorie malnutrition	2.0%	27.3%	21	Protein-calorie malnutrition	5.2%	27.4%
7804	Dizziness and giddiness	0.5%	6.4%	179	Minor symptoms, signs, findings	33.5%	17.6%
70703	Pressure ulcer, lower back	1.7%	30.8%	160	Pressure pre-ulcer skin changes or unspecified stage	2.9%	28.4%
Hx_1985	Secondary malignant neoplasm of bone and bone marrow	0.8%	22.1%	8	Metastatic cancer and acute leukemia	1.9%	20.7%

© 2019 Krumholz HM et al. *JAMA Network Open.*

eTable 3. (Continued)

ICD-9-CM				v22 HCC			
**Code**	**Description**	**Frequency of occurrence**	**Mortality rate**	**Code**	**Description**	**Frequency of occurrence**	**Mortality rate**
40291	Unspecified hypertensive heart disease with heart failure	2.2%	7.0%	85	Congestive heart failure	87.0%	11.8%
42831	Acute diastolic heart failure	7.2%	8.1%	85	Congestive heart failure	87.0%	11.8%
5849	Acute kidney failure, unspecified	21.0%	18.2%	135	Acute renal failure	21.9%	18.5%
2762	Acidosis	3.5%	23.8%	24	Disorders of fluid/electrolyte/acid-base balance	28.6%	16.5%
2767	Hyperpotassemia	5.8%	22.0%	24	Disorders of fluid/electrolyte/acid-base balance	28.6%	16.5%
51881	Acute respiratory failure	12.9%	17.6%	84	Cardio-respiratory failure and shock	29.4%	16.6%
27651	Dehydration	1.9%	22.8%	24	Disorders of fluid/electrolyte/acid-base balance	28.6%	16.5%
490	Bronchitis, not specified as acute or chronic	0.9%	7.8%	118	Other respiratory disorders	18.4%	9.40%
70705	Pressure ulcer, buttock	0.7%	26.9%	160	Pressure pre-ulcer skin changes or unspecified stage	2.90%	28.4%
7830	Anorexia	0.7%	25.2%	178	Major symptoms, abnormalities	31.4%	13.4%
79311	Solitary pulmonary nodule	0.9%	7.8%	179	Minor symptoms, signs, findings	33.5%	17.6%
78720	Dysphagia, unspecified	2.3%	24.8%	178	Major symptoms, abnormalities	31.4%	13.4%
78959	Other ascites	1.7%	20.0%	178	Major symptoms, abnormalities	31.4%	13.4%
5852	Chronic kidney disease, Stage II (mild)	1.6%	9.9%	139	Chronic kidney disease, mild or unspecified (Stages 1-2 or unspecified)	35.7%	13.5%

© 2019 Krumholz HM et al. *JAMA Network Open.*

eTable 3. (Continued)

ICD-9-CM				v22 HCC			
**Code**	**Description**	**Frequency of occurrence**	**Mortality rate**	**Code**	**Description**	**Frequency of occurrence**	**Mortality rate**
49122	Obstructive chronic bronchitis with acute bronchitis	1.6%	8.8%	111	Chronic obstructive pulmonary disease	34.7%	12.6%
5730	Chronic passive congestion of liver	0.7%	20.6%	31	Other hepatitis and liver disease	1.70%	15.9%
4011	Benign hypertension	1.8%	8.6%	95	Hypertension	37.4%	9.20%
Hx_28522	Anemia in neoplastic disease	0.7%	21.9%	49	Iron deficiency and other/unspecified anemias and blood disease	52.9%	13.6%
41072	Subendocardial infarction, subsequent episode of care	0.7%	19.0%	87	Unstable angina and other acute ischemic heart disease	3.80%	13.9%
71536	Osteoarthrosis, localized, not specified whether primary or secondary, lower leg	0.7%	8.0%	42	Osteoarthritis of hip or knee	1.30%	8.30%
00845	Intestinal infection due to Clostridium difficile	0.6%	23.3%	36	Peptic ulcer, hemorrhage, other specified gastrointestinal disorders	3.20%	16.0%
Hx_1991	Other malignant neoplasm without specification of site	0.8%	20.6%	12	Breast, prostate, and other cancers and tumors	8.60%	13.0%
7812	Abnormality of gait	1.4%	10.7%	178	Major symptoms, abnormalities	31.4%	13.4%
2630	Malnutrition of moderate degree	0.9%	22.9%	21	Protein-calorie malnutrition	5.20%	27.4%

The above ranked list contains only the individual ICD-9-CM codes that were chosen by the variable selection method. Of the non-diagnosis-based variables that were included for consideration (age, sex, history of percutaneous coronary intervention procedure, and history of coronary artery bypass grafting procedure), the method selected only age and sex.

A prepended “Hx_” on the ICD-9-CM or on the mapped v22 HCC code label indicates that it occurred during the 12-prior claim history; otherwise, the code is from the index admission.

ICD-9-CM, International Classification of Diseases, Ninth Revision, Clinical Modification; v22 HCC, Version 22 Hierarchical Condition Categories

© 2019 Krumholz HM et al. *JAMA Network Open.*

eTable 4. Top 50 *ICD-9-CM* Codes Selected by the Individual-Codes Model for Pneumonia Compared With Their Corresponding Version 22 HCC Codes

**Pneumonia**<br>[Full Cohort Mortality Rate: 16.1%]							
**ICD-9-CM**				**v22 HCC**			
**Code**	**Description**	**Frequency**	**Mortality rate**	**Code**	**Description**	**Frequency**	**Mortality rate**
1977	Malignant neoplasm of liver, secondary	0.9%	47.4%	8	Metastatic cancer and acute leukemia	3.2%	40.3%
V4986	Do not resuscitate status	17.2%	34.6%	179	Minor symptoms, signs, findings	40.7%	22.4%
49392	Asthma, unspecified type, with (acute) exacerbation	0.9%	4.0%	113	Asthma	3.8%	8.1%
1629	Malignant neoplasm of bronchus and lung, unspecified	2.3%	35.8%	9	Lung and other severe cancers	4.7%	31.2%
1970	Secondary malignant neoplasm of lung	0.8%	40.9%	8	Metastatic cancer and acute leukemia	3.2%	40.3%
78959	Other ascites	0.5%	38.0%	178	Major symptoms, abnormalities	38.3%	18.9%
4660	Acute bronchitis	1.2%	6.4%	118	Other respiratory disorders	14.6%	12.2%
51881	Acute respiratory failure	14.6%	27.5%	84	Cardio-respiratory failure and shock	35.0%	21.1%
2760	Hyperosmolality	3.6%	43.9%	24	Disorders of fluid/electrolyte/acid-base balance	39.6%	20.2%
51884	Acute and chronic respiratory failure	7.6%	21.8%	84	Cardio-respiratory failure and shock	35.0%	21.1%
490	Bronchitis, not specified as acute or chronic	0.8%	7.6%	118	Other respiratory disorders	14.6%	12.2%
7837	Adult failure to thrive	3.3%	40.4%	178	Major symptoms, abnormalities	38.3%	18.9%
27542	Hypercalcemia	0.6%	33.1%	26	Other endocrine/metabolic/nutritional disorders	30.6%	16.0%
78659	Other chest pain	0.8%	5.9%	178	Major symptoms, abnormalities	38.3%	18.9%

© 2019 Krumholz HM et al. *JAMA Network Open.*

eTable 4. (Continued)

ICD-9-CM				v22 HCC			
Code	Description	Frequency of occurrence	Mortality rate	Code	Description	Frequency of occurrence	Mortality rate
5845	Acute kidney failure with lesion of tubular necrosis	1.0%	35.0%	135	Acute renal failure	18.1%	24.2%
261	Nutritional marasmus	0.6%	38.4%	21	Protein-calorie malnutrition	10.9%	32.1%
5070	Pneumonitis due to inhalation of food or vomitus	18.8%	29.8%	114	Aspiration and specified bacterial pneumonias	7.4%	29.3%
41519	Other pulmonary embolism and infarction	0.6%	29.5%	107	Vascular disease with complications	1.0%	27.7%
262	Other severe protein-calorie malnutrition	2.7%	37.4%	21	Protein-calorie malnutrition	10.9%	32.1%
7994	Cachexia	2.1%	38.4%	21	Protein-calorie malnutrition	10.9%	32.1%
7804	Dizziness and giddiness	0.6%	7.1%	179	Minor symptoms, signs, findings	40.7%	22.4%
41071	Subendocardial infarction, initial episode of care	1.8%	31.9%	86	Acute myocardial infarction	2.0%	33.2%
Hx_1983	Secondary malignant neoplasm of brain and spinal cord	0.6%	42.8%	8	Metastatic cancer and acute leukemia	4.4%	32.2%
1985	Secondary malignant neoplasm of bone and bone marrow	1.2%	40.1%	8	Metastatic cancer and acute leukemia	3.2%	40.3%
49122	Obstructive chronic bronchitis with acute bronchitis	1.5%	9.7%	111	Chronic obstructive pulmonary disease	39.3%	14.9%
4010	Malignant hypertension	0.7%	11.6%	95	Hypertension	49.6%	14.0%
7812	Abnormality of gait	1.6%	12.0%	178	Major symptoms, abnormalities	38.3%	18.9%
79311	Solitary pulmonary nodule	1.4%	8.3%	179	Minor symptoms, signs, findings	40.7%	22.4%
5789	Hemorrhage of gastrointestinal tract, unspecified	0.8%	30.4%	36	Peptic ulcer, hemorrhage, other specified gastrointestinal disorders	4.4%	22.9%

© 2019 Krumholz HM et al. *JAMA Network Open.*

eTable 4. (Continued)

ICD-9-CM				v22 HCC			
**Code**	**Description**	**Frequency of occurrence**	**Mortality rate**	**Code**	**Description**	**Frequency of occurrence**	**Mortality rate**
2762	Acidosis	4.9%	29.7%	24	Disorders of fluid/electrolyte/acid-base balance	39.6%	20.2%
7802	Syncope and collapse	0.9%	9.7%	178	Major symptoms, abnormalities	38.3%	18.9%
5852	Chronic kidney disease, Stage II (mild)	1.0%	14.2%	139	Chronic kidney disease, mild or unspecified (Stages 1-2 or unspecified)	20.2%	18.5%
Hx_51631	Idiopathic pulmonary fibrosis	0.7%	24.2%	112	Fibrosis of lung and other chronic lung disorders	10.2%	18.7%
2639	Unspecified protein-calorie malnutrition	4.0%	31.5%	21	Protein-calorie malnutrition	10.9%	32.1%
481	Pneumococcal pneumonia [Streptococcus pneumoniae pneumonia]	1.2%	9.0%	115	Pneumococcal pneumonia, empyema, lung abscess	1.4%	15.1%
2767	Hyperpotassemia	3.7%	28.1%	24	Disorders of fluid/electrolyte/acid-base balance	39.6%	20.2%
78791	Diarrhea	2.6%	11.9%	38	Other gastrointestinal disorders	36.9%	14.3%
Hx_V440	Tracheostomy status	0.8%	16.5%	82	Respirator dependence/tracheostomy status	1.2%	17.6%
7866	Swelling, mass, or lump in chest	1.1%	17.8%	178	Major symptoms, abnormalities	38.3%	18.9%
Hx_23875	Myelodysplastic syndrome, unspecified	1.2%	24.6%	46	Severe hematological disorders	1.9%	23.0%
70720	Pressure ulcer, unspecified stage	0.8%	34.8%	160	Pressure pre-ulcer skin changes or unspecified stage	5.2%	33.3%
5533	Diaphragmatic hernia without mention of obstruction or gangrene	3.0%	12.6%	38	Other gastrointestinal disorders	36.9%	14.3%

© 2019 Krumholz HM et al. *JAMA Network Open.*

eTable 4. (Continued)

ICD-9-CM				v22 HCC			
Code	Description	Frequency of occurrence	Mortality rate	Code	Description	Frequency of occurrence	Mortality rate
515	Postinflammatory pulmonary fibrosis	3.4%	19.9%	112	Fibrosis of lung and other chronic lung disorders	6.0%	16.8%
42832	Chronic diastolic heart failure	5.0%	15.5%	85	Congestive heart failure	35.7%	19.6%
Hx_3485	Cerebral edema	0.5%	32.2%	80	Coma, brain compression/anoxic damage	1.0%	27.0%
78097	Altered mental status	2.1%	23.4%	179	Minor symptoms, signs, findings	40.7%	22.4%
49322	Chronic obstructive asthma with (acute) exacerbation	2.1%	9.8%	111	Chronic obstructive pulmonary disease	39.3%	14.9%
4940	Bronchiectasis without acute exacerbation	1.3%	10.7%	112	Fibrosis of lung and other chronic lung disorders	6.0%	16.8%
V5861	Long-term (current) use of anticoagulants	10.3%	13.1%	191	Post-surgical states/aftercare/elective	48.7%	14.2%
4589	Hypotension, unspecified	3.7%	24.3%	109	Other circulatory disease	7.5%	20.0%

The above ranked list contains only the individual ICD-9-CM codes that were chosen by the variable selection method. Of the non-diagnosis-based variables that were included for consideration (age, sex, history of percutaneous coronary intervention procedure, and history of coronary artery bypass grafting procedure), the method selected only age and sex.

A prepended "Hx_" on the ICD-9-CM or on the mapped v22 HCC code label indicates that it occurred during the 12-prior claim history; otherwise, the code is from the index admission.

ICD-9-CM, International Classification of Diseases, Ninth Revision, Clinical Modification; v22 HCC, Version 22 Hierarchical Condition Categories

© 2019 Krumholz HM et al. *JAMA Network Open.*

eTable 5. Centers for Medicare & Medicaid Services Publicly Reported Performance Categories for the CMS Model Compared With the Individual-Codes Model for 30-Day Mortality Measures Among Hospitals With at Least 25 Cases

A. Acute myocardial infarction performance bucket shift

**CMS model buckets**	**Individual-codes model buckets**			
	**Better than the national rate**	**No different than the national rate**	**Worse than the national rate**	**Total**
**Better than the national rate**	20	10	0	30
**No different than the national rate**	14	2143	18	2175
**Worse than the national rate**	0	5	8	13
**Total**	34	2158	26	2218

B. Heart failure performance bucket shift

**CMS model buckets**	**Individual-codes model buckets**			
	**Better than the national rate**	**No different than the national rate**	**Worse than the national rate**	**Total**
**Better than the national rate**	86	38	0	124
**No different than the national rate**	99	2990	50	3139
**Worse than the national rate**	0	29	31	60
**Total**	185	3057	81	3323

C. Pneumonia performance bucket shift

**CMS model buckets**	**Individual-codes model buckets**			
	**Better than the national rate**	**No different than the national rate**	**Worse than the national rate**	**Total**
**Better than the national rate**	167	25	0	192
**No different than the national rate**	323	2968	209	3500
**Worse than the national rate**	2	84	99	185
**Total**	492	3077	308	3877

These tables show the individual-codes model shifting more hospitals away from the middle bucket, which can be associated with better discrimination of hospital performance.
CMS, Centers for Medicare & Medicaid Services

© 2019 Krumholz HM et al. *JAMA Network Open.*
